# An Online Influenza Surveillance System for Primary Care Workers in Switzerland: Observational Prospective Pilot Study

**DOI:** 10.2196/17242

**Published:** 2020-09-10

**Authors:** Sébastien Martin, Muriel Nirina Maeder, Ana Rita Gonçalves, Baptiste Pedrazzini, Jean Perdrix, Carine Rochat, Nicolas Senn, Yolanda Mueller

**Affiliations:** 1 Center for Primary Care and Public Health (Unisanté), Lausanne Department of Family Medicine University of Lausanne Lausanne Switzerland; 2 Laboratory of Virology, Division of Infectious Diseases National Reference Centre of Influenza Geneva University Hospitals Geneva Switzerland; 3 Center for Primary Care and Public Health (Unisanté) Department of Ambulatory Care and Community Medicine University of Lausanne Lausanne Switzerland

**Keywords:** influenza, surveillance system, primary care, online, nosocomial, transmission

## Abstract

**Background:**

A better understanding of the influenza epidemiology among primary care workers could guide future recommendations to prevent transmission in primary care practices. Therefore, we designed a pilot study to assess the feasibility of using a work-based online influenza surveillance system among primary care workers. Such an approach is of particular relevance in the context of the coronavirus disease (COVID-19) pandemic, as its findings could apply to other infectious diseases with similar mechanisms of transmission.

**Objective:**

This study aims to determine the feasibility of using a work-based online influenza surveillance system for primary care workers in Switzerland.

**Methods:**

Physicians and staff of one walk-in clinic and two selected primary care practices were enrolled in this observational prospective pilot study during the 2017-2018 influenza season. They were invited to record symptoms of influenza-like illness in a weekly online survey sent by email and to self-collect a nasopharyngeal swab in case any symptoms were recorded. Samples were tested by real-time polymerase chain reaction for influenza A, influenza B, and a panel of respiratory pathogens.

**Results:**

Among 67 eligible staff members, 58% (n=39) consented to the study and 53% (n=36) provided data. From the time all participants were included, the weekly survey response rate stayed close to 100% until the end of the study. Of 79 symptomatic episodes (mean 2.2 episodes per participant), 10 episodes in 7 participants fitted the definition of an influenza-like illness case (attack rate: 7/36, 19%). One swab tested positive for influenza A H1N1 (attack rate: 3%, 95% CI 0%-18%). Swabbing was considered relatively easy.

**Conclusions:**

A work-based online influenza surveillance system is feasible for use among primary care workers. This promising methodology could be broadly used in future studies to improve the understanding of influenza epidemiology and other diseases such as COVID-19. This could prove to be highly useful in primary care settings and guide future recommendations to prevent transmission. A larger study will also help to assess asymptomatic infections.

## Introduction

Available estimates suggest that 5%-20% of the global population is affected by influenza annually [1]. In Europe, seasonal influenza epidemics have the largest disease burden among all communicable disease in terms of disability-adjusted life years, mainly because of their large contribution to premature mortality [2].

Primary care physicians play a key role during seasonal influenza epidemics, even though most individuals presenting influenza-like symptoms do not seek a medical consultation [3]. In Switzerland, for example, influenza-like illnesses drive 1.4%-3.4% of the general population to consult a primary care physician per influenza season [4]. In addition, primary care physicians are responsible for vaccinating the population, especially vulnerable groups such as older patients and patients with comorbidities. Finally, primary care physicians are at the epicenter of the influenza sentinel networks that exist in many countries, which are used by public health authorities to officially declare national influenza epidemics.

Primary care physicians and staff working in primary care practices (collectively referred to as “primary care workers” hereafter) have a central role in patient care during the seasonal epidemics. For these reasons, they could potentially play a role in the influenza transmission chain. Indeed, primary care physicians were shown to have high levels of influenza antibody titers [5], and health care workers, in general, are at higher risk of influenza compared to adults working in non–health care settings [6-8].

However, the role of primary care practices in the transmission chain is largely unknown. Patients visiting the emergency department during the influenza season were found to have a higher risk of influenza-like illnesses compared with community controls [9]. Similarly, children visiting a pediatric clinic were at increased risk of presenting with influenza-like illnesses in the following days [10]. To prevent nosocomial transmission of influenza, vaccination of health care workers is recommended, and there is some evidence of the effectiveness of this strategy in preventing influenza infection among primary care physicians [11]. However, most of the work on nosocomial influenza has been conducted in hospitals or long-term care facilities [8,12,13]. Indeed, the data on the epidemiology of influenza among health care workers is very rarely described, particularly in primary care practices. Furthermore, evidence on interventions that reduce influenza transmission in primary care practices is particularly scarce [14]. Therefore, a better understanding of influenza epidemiology among primary care workers could guide future recommendations to prevent influenza transmission in this setting. This issue is of particular interest in contemporary times, as it also concerns other infectious diseases with transmission mechanisms similar to those of influenza, such as the coronavirus disease (COVID-19), for which transmission by primary care workers could play an important role.

The wide availability of the internet and the growth of digital communication technologies has led to the increasing use of these resources in public health surveillance. Online systems to monitor the activity of influenza in the general population have been developed previously, based on data provided by volunteers who self-report their symptoms via the internet throughout the influenza season [15,16]. More recently, such systems have included self-swabbing from participants [17,18]. The interest and feasibility of such an online system among health care workers are being evaluated in a hospital setting, but no results have been published to date [19].

As part of a longer-term national project to clarify the role of primary care practices in the transmission of influenza, we conducted a pilot study to assess the feasibility of a prospective work-based online influenza surveillance system among primary care workers [9]. The main objectives of this pilot study were to assess the participation of primary care workers in a weekly online influenza surveillance system as well as to examine the sustainability and feasibility of self-administration of nasopharyngeal swabs among study participants in such a system. We also monitored the influenza-like illnesses attack rate and the confirmed influenza cases over the entire influenza season of 2017-2018 among primary care workers.

## Methods

We conducted a prospective observational study in three medical centers: one public walk-in clinic in Lausanne and two private family medicine practices purposively selected due to their regular collaboration with our department.

### Recruitment

Data collection took place from October 2017 to April 2018. The study population corresponded to the primary care workers active in the medical centers during the pilot study. Inclusion criteria for medical centers included any family medicine practices or walk-in clinics providing primary care in the canton of Vaud, that were willing to participate in the project. For individual participants, inclusion criteria were age≥18 years and the presence of an employment contract during the study period. Members of the Swiss influenza sentinel medical practice network (Sentinelled) were excluded as participant centers, and staff members without contact with patients were excluded as individual participants. Enrollment was open between the beginning of the influenza surveillance season in Switzerland (week 40) and the beginning of the influenza epidemic as declared by the national influenza surveillance system (week 51 in the 2017-2018 season).

The staff was invited to participate in the survey by one of the medical center’s head physicians (center manager). After receiving a numbered information sheet, each staff member was asked to provide information about the inclusion criteria; respond about their intention to participate in the study; and, if applicable, sign an informed consent form. This action was reinforced by verbal reminders during a team meeting. For this pilot study, a convenience sample of 50 subjects was considered appropriate.

**Figure 1:**
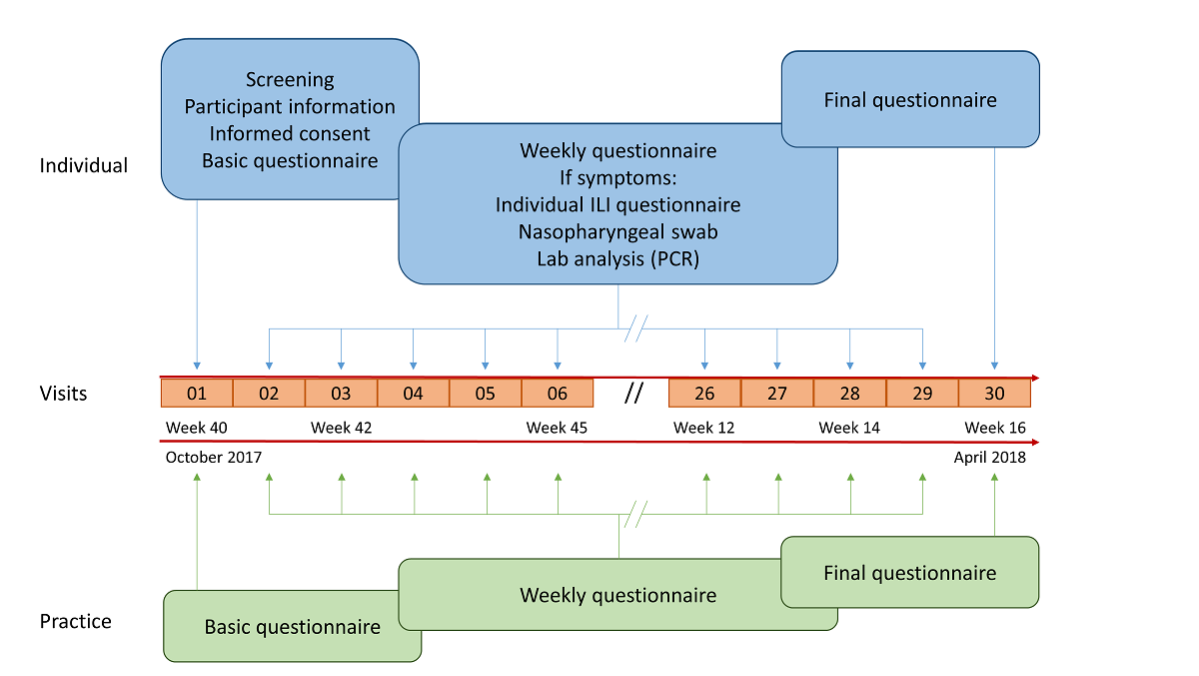
Outline of the study. ILI: influenza-like illness; PCR: polymerase chain reaction.

The outline of the study is presented in Figure 1. The center manager had to complete a basic questionnaire about the number of employees, their duties, activity rate, and contacts with patients. He was also asked to provide information about staff vaccination and the use of other preventive measures at the practice (handwashing and disinfection, mask-wearing, isolation of patients, frequency of disinfection, and ventilation of waiting room). He then had to answer a weekly questionnaire about the number absent days of the staff, the number of vaccinations among staff since the previous week, the changes in preventive measures, and the number of patients with influenza syndrome seen daily as a proportion of the total number of patients visiting during the previous week. At the end of the study, he was asked to answer a final questionnaire about the feasibility of the weekly questionnaire.

**Figure 2:**
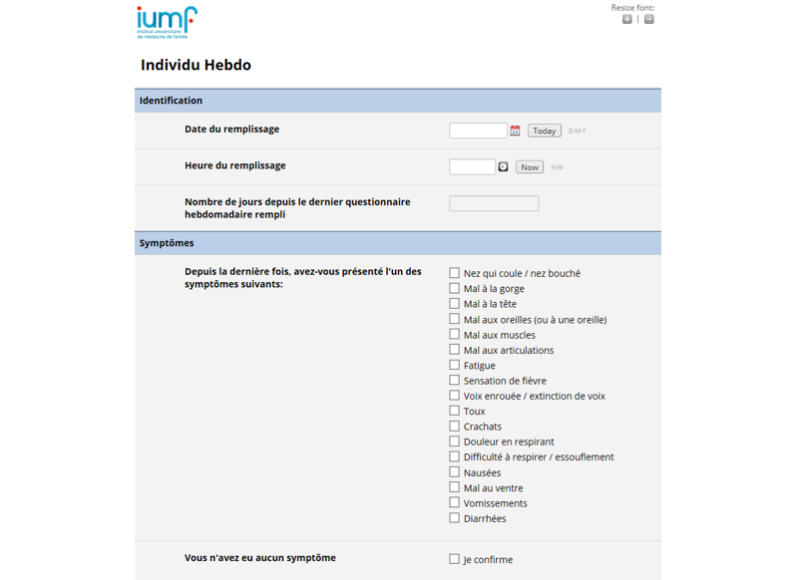
Weekly online questionnaire for study participants.

The study participants were asked to complete a basic questionnaire about demographics, their function at the study site, type of contacts with patients, and compliance with vaccination protocols and other preventive measures. Subsequently, they had to answer a weekly questionnaire about influenza-like illnesses symptoms during the past week (or since the last completed questionnaire if the previous week’s data were missing), similar to the one used by the Vinylbenzene questionnaire [16,20] (Figure 2). In the case of influenza-like illnesses symptoms, defined by a history of fever, usually with acute onset (temperature>38 °C), and a cough or sore throat, the participants were asked to provide the exact symptoms start date and the number of missed working days. They were then invited to perform a nasopharyngeal swab and asked about the tolerance and feasibility of a self-administered swab. At the end of the study, they were asked to answer a final questionnaire about the feasibility of the weekly questionnaire, estimated time required to complete it, and suggestions for improving the study procedures. Finally, they were asked about their willingness to perform a serological test for influenza at the beginning and end of the investigation or to conduct a self-administered nasopharyngeal swab in the absence of symptoms.

Data were collected online using RedCap (Research Electronic Data Capture) software (Vanderbilt University), with a link sent to participants by email every Monday. The link was sent to the participant’s private or professional email address according to their preference. The questionnaire could be completed until the following Friday. One email reminder was sent if the questionnaire was not completed after 3 days. Participants with influenza-like illnesses symptoms who did not provide a nasopharyngeal swab within 3 weeks were asked about their reasons for not performing a nasopharyngeal swab. The evaluation of missing results and interrupted follow-ups was an integral part of the pilot study.

Nasopharyngeal swabs were sent to the National Reference Centre of Influenza (Geneva, Switzerland). They were tested weekly for influenza A and B by reverse transcriptase real-time polymerase chain reaction (rRT-PCR). Twice during the season, on weeks 3 and 16, samples were tested by rRT-PCR for a panel of respiratory pathogens including influenza A; influenza A (subtype H1N1); influenza B; rhinovirus; coronavirus species NL63, 229E, OC43, and HKU1; parainfluenza 1-4; human metapneumovirus A/B; bocavirus; respiratory syncytial virus A/B; adenovirus; enterovirus; parechovirus; and *Mycoplasma pneumoniae*.

To preserve participants’ privacy, no participants’ study data were provided to center managers. This point was specified in the participants’ information sheet. The study was conducted in accordance with the principles of the Helsinki Declaration. Each study participant signed an informed consent form, and the human research ethics committee of the canton of Vaud approved the study (CER-VD2017-01519).

### Statistical Analysis

Data were analyzed using Stata 13 statistical software (StataCorp). Results for primary and secondary outcomes were presented as proportion, incidence rates, and attack rates for the total study population. The chi-square and Wilcoxon rank-sum tests were used to compare proportions and medians, respectively, between categories (practices, professions, auto- vs hetero-swab). The significance level was set at *P* value<.05.

## Results

### Participation

**Figure 3:**
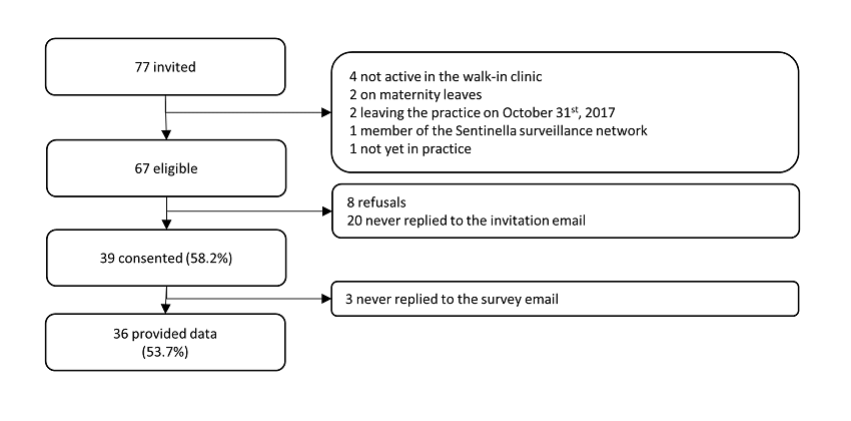
Participant flow chart, feasibility study of an online influenza surveillance system among primary care workers of three clinics in Switzerland, 2017-2018.

A total of 77 primary care workers from the three centers were invited to participate in our study. Of the 67 eligible persons based on the inclusion and exclusion criteria, 39 (58%) participants, comprising 22 physicians and 17 medical assistants, consented to the study. The distribution of 39 participants was as follows: 19 of the available 47 (40%) primary care workers from the walk-in clinic and 20 of the available 20 (100%) from two private practices enrolled in the study. Of the 39 participants, 36 (92%) finally provided data (Figure 3). All 28 who did not consent to the study were working in the walk-in clinic. The distribution of nonparticipants corresponded to 10 of the eligible 18 (55%) external medical supervisors, 10 of the 14 (71%) physicians in postgraduate training, 8 of the 15 (53%) eligible medical assistants, and 5 of the 5 (100%) eligible secretaries. The mean age of participants was 42.2 (SD 12.1) years, and 22 (61%) participants were women. The median number of years working in the same practice was 3 (range 2-12 years). In total, 22/36 (61%; missing 1 participant’s data) participants were vaccinated against influenza. The proportion of participants that were vaccinated was 18/21 (85%) for physicians and 4/14 (28%, 1 missing data point) for medical assistants (χ^2^_1_=11.7; *P*=.001).

### Feasibility

**Figure 4:**
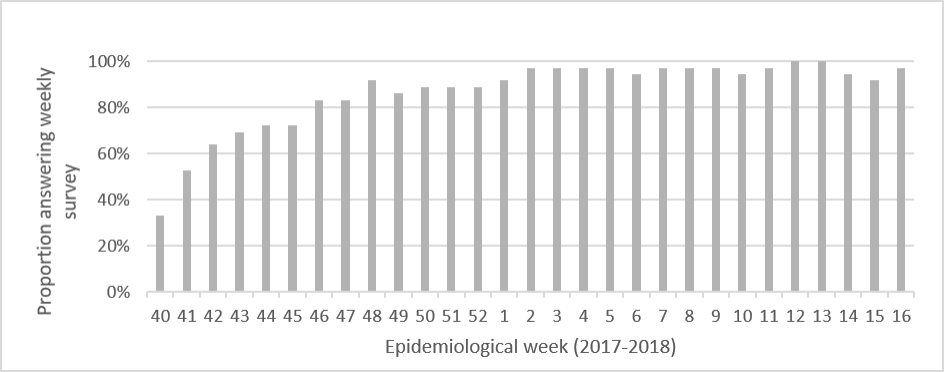
Proportions of participants answering to weekly online survey (n=36).

The proportion of workers responding to the weekly online survey per week increased during the first weeks of the study with progressive enrollment. From the time all participants were included, the response rate stayed close to 100% until the end of the study (Figure 4). Among the 36 participants, 25 (69%) primary care workers completed at least 80% of the weekly online surveys.

In the context of survey responses, 23 (63%) participants answered the questionnaires mainly at work, and 12 (33.3%) mainly at home. In terms of access, 20 (55.6%) participants accessed the surveys using the workplace computer, 8 (22%) accessed them through their smartphone, and 7 (19%) accessed them through their private computer. The median time to complete the initial individual questionnaire was 3.0 (IQR 2.0-4.0) minutes and the median time to complete the final individual questionnaire was 2.5 (IQR 2.0-4.0) minutes. Completing the weekly survey took 10 seconds on average in the absence of symptoms and 2 minutes and 17 seconds in case of symptoms. All participants completed questionnaires until the end.

### Acceptability of Swabbing

Of the 15 nasopharyngeal swabs (2 pharyngeal, 3 with missing data), 6 (40.0%) were autoswabs, of which 5 were performed by physicians and 1 by a medical assistant. The remaining were heteroswabs. In terms of preference for methods, 9 of 10 medical assistants preferred a heteroswab instead of an autoswab. The median depth of the swab was 8.5 cm (range 6-10 cm, 2 participants with missing data). Two participants (13.3%) mentioned nonsevere adverse effects of the swab (itchy nose, burning sensation). Median discomfort was estimated at 6 on a 10-point pain scale (IQR 4-8, n=15). The level of discomfort was significantly higher among medical assistants than among physicians (median 7.5, IQR 6.5-8, vs median 4, IQR 3-4; rank-sum *P*=.02), whereas no significant difference in discomfort was observed between heteroswabs and autoswabs (median 7, IQR 4-8 vs median 4, IQR 3-7; rank-sum *P*=.13).

**Figure 5:**
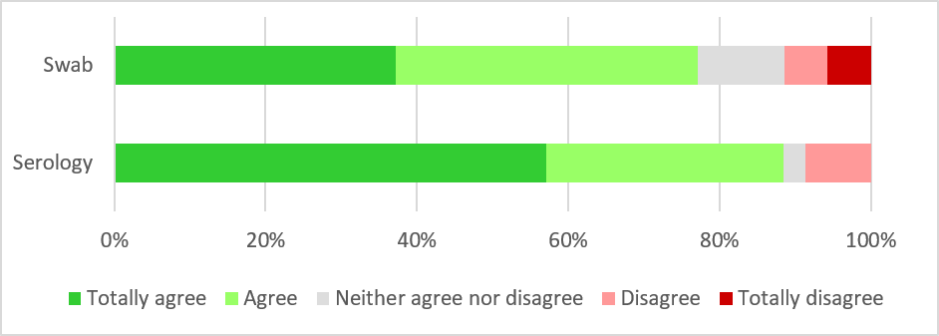
Willingness to perform a serological test or a self-administered nasopharyngeal swab in the absence of symptoms (n=35).

Overall, swabbing was considered relatively easy and most participants “agreed” or “totally agreed” that swabbing explanations were clear and sufficient (Figure 5).

The main reasons for not performing a swab in case of influenza-like symptoms were that participants believed that symptoms were too light or already over (n=21), the diagnosis was not influenza (n=17), they already had taken a swab for that episode (n=5), a swab was unnecessary (n=3), a swab would be negative (n=1), or their symptoms did not fit the influenza-like illnesses case definition (n=1).

### Attack Rate of Influenza

Of 79 symptomatic episodes (mean 2.2 per participant), 10 fitted the influenza-like illnesses case definition. Five participants said that they missed work for an average of 2 (range 1-5) days, and 9 participants reduced their daily activities for an average of 3.3 (range 1-10) days. For 31 of the 79 (39%) episodes, participants said they had worked without difference by practice while having symptoms of fever, sore throat, or cough (χ^2^_3_=2.2; *P*=.53).

In total, 20 swabs were performed for 19 symptomatic episodes (2 swabs for the same influenza A episode) occurring in 16 participants, including 8 of the 10 influenza-like illnesses episodes. The swabs were performed a median of 3 days after the start of symptoms (IQR 1-5) and were received in the lab a median of 2 (IQR 1-3) days later. More than half of the initial symptomatic episodes were swabbed (15/29, 51%), compared to only 10% of the subsequent 40 episodes (4/40, 10%).

A virus was identified in 10 of 19 (52%) symptomatic episodes and 4 of 8 (50%) influenza-like illnesses episodes, respectively. One swab was positive for influenza A H1N1 (attack rate: 2.8%, 95% CI 0.4%-18.3%; Table 1). In addition, 2 cases of coronaviruses HKU1 were identified among the 12 swabbed participants with symptoms not fitting the influenza-like illnesses case definition (data not shown).

**Figure 6:**
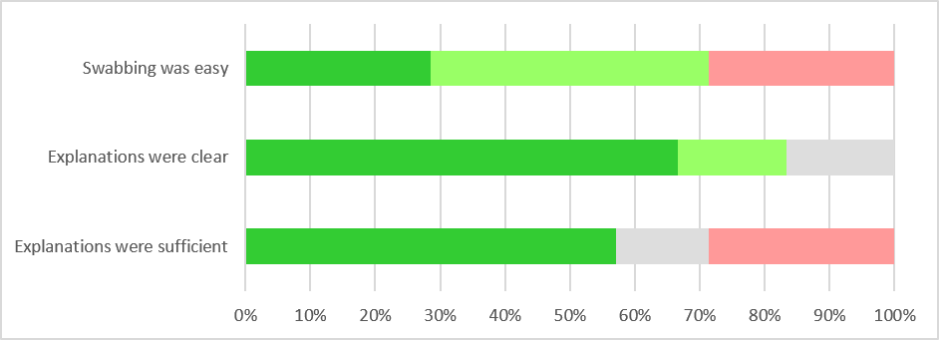
Acceptability of nasopharyngeal swab (n=15).

In case of a future study that would also target asymptomatic influenza, most participants “agreed” or “totally agreed” to perform a serological test for influenza at the beginning and end of the surveillance season or to self-administer a nasopharyngeal smear in the absence of symptoms (Figure 6). Participants preferred serological tests over other methods (54% vs 28%; for no preference: 17%).

**Table 1:** Clinical characteristics and swab results of 10 influenza-like illness episodes reported by practice staff.

Month	Temperature (°C)	Fever history	Sore throat	Cough	Swab	Result
November 2017	38	Yes	Yes	No	Yes	Negative
November 2017	37	Yes	Yes	Yes	Yes	Rhinovirus
December 2017	37	Yes	Yes	Yes	Yes	Coronavirus OC43
January 2018	N/A^a^	Yes	No	Yes	Yes	Negative
January 2018	37	Yes	Yes	Yes	Yes	Influenza A H1N1
January 2018	39	Yes	Yes	Yes	No	N/A
February 2018	N/A	Yes	Yes	Yes	Yes	Negative
February 2018	38	No	Yes	No	Yes	Negative
February 2018	38	Yes	Yes	Yes	Yes	Rhinovirus
February 2018	37	Yes	Yes	Yes	No	N/A

^a^N/A: not applicable.

## Discussion

### Principal Results

To prevent influenza transmission to vulnerable patients consulted in primary care practices, it is important to understand the influenza epidemiology among primary care workers. Although surveillance studies among cohorts of health care workers have been conducted in hospital settings, our pilot study is the first one to set up a prospective online influenza surveillance system among the staff of primary care practices. It successfully demonstrated the feasibility of a work-based online influenza surveillance system combined with self-administrated nasopharyngeal swabs in participants with influenza-like illnesses. It also provided detailed information about the feasibility and the level of participation of primary care workers in such a surveillance system. Primary care workers were willing to participate in such a system, with more than half of all eligible workers giving their consent and providing data. Maintaining a sufficient level of participation over time is an important factor in guaranteeing the representativeness of a monitoring system. In our study, almost all participants that provided initial consent maintained their participation and the majority completed most of the weekly online surveys. Furthermore, few nonserious side effects of nasopharyngeal swabbing were mentioned. The discomfort was acceptable, and swabbing was considered relatively easy regardless of the swabbing procedure (autoswab or heteroswab).

Participation was much better in smaller private practices than in the public walk-in patient clinic. Moreover, participation was better among regular staff than among rotating staff (external medical supervisors and physicians in postgraduate training) and administrative staff (secretaries), suggesting that permanent staff are more readily involved in a research project that extends over several months than rotating staff. These findings can also be related to the size of the facility, which allows for more personalized contacts with participants when presenting the study and answering their questions. From the perspective of a future larger-scale study in Switzerland, this observation represents an advantage, as the size of the two practices included in the study closely matches the size of the majority of Swiss primary care practices.

The time spent in providing data and the impact on the privacy of participants can influence the feasibility as well as the participation in a surveillance system. In our study, the median time needed to complete the questionnaires was noticeably short. In addition, most of the participants answered the questionnaires on their workplace and accessed the surveys using the workplace computer.

During an influenza episode, the affected person can be expected to stay at home. For this reason, we decided to distribute swab material to all participants and gave them the freedom to either self-administer the swab or ask a colleague to administer it to them when they fitted the influenza-like illnesses case definition, thereby maximizing the chances of a swab to be performed when indicated. Providing a choice to perform an autoswab or a heteroswab was an effective strategy, as nearly half of the smears taken were autoswabs that did not seem to have caused any problems for physicians, while a little more than half were heteroswabs, mainly among medical assistants. To maximize the number of swabs performed by participants and to study their feasibility under the best possible conditions, we used a less restrictive influenza-like illness case definition than official definitions [21]. In some cases, participants waived the need to perform a swab even if the criteria were met, mostly because they judged it unnecessary, or because they had already taken a swab previously. These results highlight the need for clearer explanations and a clearer framework about the indication for a swabbing in further studies.

In such a study, addressing the issue of privacy is important, as it could lead to underreported episodes by participants because of the risk of being absent from work or being penalized for not staying home in case of symptoms. For this reason, it was made clear to participants that employers were not receiving any information about their employees’ study data. During terminal team meetings at the end of the study conducted to present and discuss the results, participants did not identify any privacy issues.

In our study, the attack rate of influenza during the 2017-2018 season was low, but considering the large confidence interval, it was within the range of that estimated by other studies conducted either in the general population or among health care workers [22,23]. This finding leads to the question of asymptomatic influenza episodes among vaccinated health care workers. Recent studies showed that a significant number of health care workers with respiratory symptoms were afebrile prior to their diagnosis and may pose a risk of influenza transmission to patients and coworkers [23]. To quantify this phenomenon, future studies should be able to assess asymptomatic infections among participants by performing either serological tests before and after the annual influenza epidemic or a self-administered nasopharyngeal swab in the absence of symptoms. The results of our study show that both serologies and swabs would be accepted by participants, with a preference for serological testing.

### Limitations

The inclusion of only three practices is not sufficiently representative. However, they are typical of the most frequent type of medical practices in Switzerland, due to their location in a suburban region and their organization as a team of doctors and medical assistants [24]. The walk-in clinic, for its part, is representative of a model of larger medical centers that are currently emerging in Switzerland. We believe that our data on participation are sufficient for planning a larger study. Finally, the size of the sample was too limited to assess the attack rate of influenza among primary care workers, but this was not the main objective of our study.

### Conclusion

A work-based online influenza surveillance system among primary care workers, combined by self-administrated nasopharyngeal swabs performed by participants, is a promising methodology for conducting a large-scale study that combines data on staff and patients. Precise estimation of the influenza attack rate among primary care workers, of both symptomatic and asymptomatic infections, will make it possible to recommend preventive measures for primary care practices. This could be immensely useful in guiding future recommendations for preventing nosocomial transmission.

In infectious diseases, including the recent COVID-19 outbreak that displays a high risk of spread with a similar nosocomial transmission, applying such a surveillance system among primary care workers could limit virus spread. Symptomatic primary care workers could be isolated quickly, limiting the risk of contamination. In the context of infectious disease where transmission by asymptomatic carriers is important, surveillance could include systematic testing to identify healthy carriers among primary care workers.

## Abbreviations

COVID-19: coronavirus disease
RedCap: Research Electronic Data Capture
rprt-PCR: real-time polymerase chain reaction
